# The Applications and Mechanisms of Superoxide Dismutase in Medicine, Food, and Cosmetics

**DOI:** 10.3390/antiox12091675

**Published:** 2023-08-27

**Authors:** Mengli Zheng, Yating Liu, Guanfeng Zhang, Zhikang Yang, Weiwei Xu, Qinghua Chen

**Affiliations:** College of Animal Science and Technology, Hunan Agricultural University, Changsha 410128, China

**Keywords:** superoxide dismutase, oxidative stress, food, medicine, regulating mechanism

## Abstract

Superoxide dismutase (SOD) is a class of enzymes that restrict the biological oxidant cluster enzyme system in the body, which can effectively respond to cellular oxidative stress, lipid metabolism, inflammation, and oxidation. Published studies have shown that SOD enzymes (SODs) could maintain a dynamic balance between the production and scavenging of biological oxidants in the body and prevent the toxic effects of free radicals, and have been shown to be effective in anti-tumor, anti-radiation, and anti-aging studies. This research summarizes the types, biological functions, and regulatory mechanisms of SODs, as well as their applications in medicine, food production, and cosmetic production. SODs have proven to be a useful tool in fighting disease, and mimetics and conjugates that report SODs have been developed successively to improve the effectiveness of SODs. There are still obstacles to solving the membrane permeability of SODs and the persistence of enzyme action, which is still a hot spot and difficulty in mining the effect of SODs and promoting their application in the future.

## 1. Introduction

Accelerated skin aging, the increased proportion of younger patients with diabetes and cancer, and the increased proportion of patients with neurodegenerative diseases such as amyotrophic lateral sclerosis and Alzheimer’s disease might be related to oxidative stress in the body [1,2,3,4]. It has been proven that many abnormal physiological and metabolic processes in organisms produce biological oxidants such as superoxide radicals (O_2_^•−^), hydroxyl radicals (•OH), and hydrogen peroxide (H_2_O_2_), which could endow several different biological targets with reactivity and instability, such as DNA, proteins, and membrane lipids [5]. In the oxidative stress state in which a large amount of biological oxidants lead to an imbalance between oxidation and anti-oxidation in vivo, cellular oxidants activate various apoptosis inducers through certain transcription factors or directly lead to lipid peroxidation, protein and DNA damage, and enzyme expression disorders. Physiological production of superoxide radical anion and hydrogen peroxide is essential for radox signaling in cells, but their excessive generation could lead to oxidative stress and oxidative modifications of biomolecules and lipid peroxidation, which could be related to pathophysiological processes. We still do not know to what extent the production of oxidants is responsible for the pathogenesis of diseases and to what extent it only accompanies them.

Superoxide dismutase (SOD) acts as an antioxidant enzyme that scavenges oxygen radicals through oxidation/reduction cycles at a very high reaction rate through transition metal ions present at the active site. SOD decomposes O_2_^•−^ into H_2_O_2_ with the release of molecular oxygen. SOD is a kind of enzyme containing Cu, Mn, Zn, and other metal ions. It is widely distributed in plants, animals, and microorganisms [6]. In the process of eliminating free radicals, SOD requires cofactors such as iron, manganese, or copper and zinc to exert maximum catalytic activity when metabolizing toxic intermediates. The metal-binding site is located between the two domains of SOD, and the side chains include aspartate, histamine, and histidine [7]. These cofactors tend to donate electrons to O_2_^•−^ and regenerate throughout the catalytic mechanism.

SOD participates in the disproportionation of superoxide free radicals inside and outside the cells so that the cell membrane and DNA are protected from damage by oxygen free radicals [8]. SOD enzymes (SODs) play an important role in balancing the concentration of biological oxidants, which can protect all aerobic organisms from oxidative damage caused by biological oxidants. SOD is one of the most antioxidant enzymes, which can catalyze O_2_^•−^ to form H_2_O_2_ to regulate the body’s signal transduction. H_2_O_2_ could be used as a second messenger to regulate a variety of signal transduction in inflammation, angiogenesis, and other processes, affecting the expression of many genes in physiological and pathological conditions. Additionally, H_2_O_2_ diffuses across the cell membrane via aquaporin aqueous channels (AQPs), transposing redox signals from the location where the redox signal is generated to the site of interest [9].

SODs, as enzymes characterized by very fast second-order constants, facilitate the encounter of enzymes with substrates during enzyme-catalyzed disproportionation reactions by the presence of a strong attraction electric field generated by the charge on the surface of the enzyme or by reducing the dimensionality of the search process (electrostatic promotes diffusion) [10]. O_2_^•−^ is usually directed to the active site of Cu/Zn-SOD by a conserved electrostatic loop containing positively charged residues such as Lys136 and Arg143. Since the rate of reaction of SOD with O_2_^•−^ is limited only by diffusion, the efficiency of SOD can be improved by electrostatic induction; that is, by enhancing the positive charge or accessibility of the active site. The rate of spontaneous non-enzymatic arrangement is relatively low (about 2 × 10^5^ M^−1^s^−1^ at physiological pH), and the superoxide dismutase catalyzed disproportionation reaction increases the rate by a factor of 10,000 [10].

Metabolic reactions that consume oxygen molecules are the main source of superoxide. O_2_^•−^ production could be increased from several cellular sites, such as the mitochondrial electron respiratory chain (this is considered the main source of O_2_^•−^, in fact, in mammals, more than 95% of the daily consumed oxygen is reduced to water in the respiratory chain, of which 1–2% is converted to O_2_^•−^ by proteins in the mitochondrial electron transport chain); NADPH oxidase NOX (NOX is membrane-bound oxidoreductases that transfer electrons from intracellular NADPH to extracellular oxygen, producing reactive oxygen species, including superoxide anions and hydrogen peroxide [11]. NADPH oxidase in macrophages produces large amounts of O_2_^•−^ defense against pathogens and other invaders [12]), cyclooxygenase, lipoxygenase, xanthine oxidase XO (XO activity is a source of superoxide anions in the extracellular space of skeletal muscle [13]), cytochrome P450 oxidoreductase/cytochrome P450 reductase, and electron transport chains, peroxisomes, nuclear membranes, and cytoplasmic membranes found in the endoplasmic reticulum, all converting O_2_ to superoxide.

Catalase (CAT), SODs, and glutathione peroxidase (GSH-Px) together constitute the body’s active oxygen scavenging system, which plays a very important role in the removal and reduction in O_2_^•−^, H_2_O_2_, and •OH. The enzymatic activity of extracellular superoxide dismutase supports superoxide disproportionation to H_2_O_2_. In addition, SOD proteins have the ability to bind to cell surface proteoglycans and extracellular matrix components in a positively-charged C-terminal region (extracellular matrix binding region). The efficacy of SODs makes them more widely used in medical, food, and skincare products. They have biological functions such as regulating the body’s redox reaction, scavenging free radicals, monitoring disease occurrence, and anti-oxidation.

This review briefly introduces the types, structures, and activity mechanisms of natural SODs. Then, based on the study of natural SODs, the catalytic mechanism and regulatory factors of SODs activity were expounded. After that, the application of SOD enzymes in various fields was summarized. Finally, the challenges and prospects in the current development are put forward.

## 2. Biological Characteristics of SOD

### 2.1. Types and Properties of SOD

The SOD (enzyme code EC1.15.1.1) family is a series of metal enzymes that scavenge superoxide anion radicals. It has the effect of anti-oxidation, which could convert superoxide into O_2_ and H_2_O_2_ through a disproportionation reaction. In addition, it prevents cells from oxidative stress damage. SODs in eukaryotes are divided into three types according to their binding metal cofactors and cellular localization, namely, copper–zinc superoxide dismutase (Cu/ZnSOD or SOD1) present in the cytoplasm or secreted into the extracellular fluid, manganese-containing superoxide dismutase (Mn-SOD or SOD_2_) present in the mitochondrial matrix, and extracellular superoxide dismutase (EC-SOD or SOD_3_) (Table 1) [6,14].

Cu/ZnSOD has long been considered the major copper-containing protein in eukaryotes, but its enzymatic function was not discovered until McCord and Fridovich discovered in 1969 that “cuprozinc amine oxidase” could dismutate superoxide [15]. SOD1 is the main intracellular SOD enzyme, widely distributed in the cytoplasm, nucleus, and cell membrane [16]. It exists as a 32 kDa homodimer, mainly confined to the cytoplasm, with a smaller proportion in the mitochondrial intermembrane space (IMS). The enzyme activity of SOD1 depends on the presence of copper and zinc, but zinc is only related to the molecular structure of the enzyme and has no catalytic activity. It involves proper protein folding and stability. The SOD activity of the re-metalated derivatives is proportional to the amount of copper binding at the natural copper site. In addition, copper cannot be replaced by another metal, while zinc can be replaced by cobalt and copper. The activity of SOD1 requires a catalytic copper to scavenge O_2_^•−^.

SOD2 is an enzyme containing mitochondrial manganese, localized in the mitochondrial matrix, consisting of a 96 kDa homotetramer [6,17]. Manganese is related to the catalytic activity of enzymes. SOD2 is synthesized in the cytoplasm and directed to the mitochondria through signal peptides, which are involved in oxygen disproportionation produced by respiratory enzyme chains. It is found in all mitochondria and cellular fluids except erythrocytes, and its molecular weight varies depending on distribution and source.

SOD3, with Cu^2+^/Zn^2+^ as the auxiliary group, belongs to Cu/Zn-SOD, which is distributed in blood, lymph, synovial fluid, and tissues. As a secreted extracellular copper/zinc-containing SOD (ecSOD), SOD3 is a 135 kDa homotetramer consisting of two disulfide-linked dimers in most species [18]. It eliminates the reactive oxygen species produced by metabolism in the body and resists the destruction of superoxide ions in the internal and external environment [19].

### 2.2. The Structure and Catalytic Function of SODs

**Figure 1:**
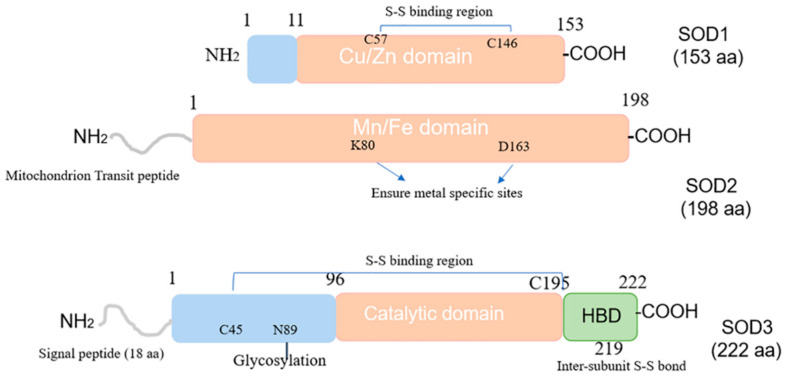
Composition of SOD1, SOD2, and SOD3. HBD: heparin-binding domain.

From the overall structure, it can be observed that SOD1 and SOD2 are composed of homotetramers of 32 kDa and 96 kDa, respectively, while SOD3 is composed of diametrically-bonded disulfides [6]. The structure of the SOD protein family is shown in Figure 1. The SOD1 protein contains two functional regions: the amino binding region includes 1–10 amino acids, and the Cu/Zn binding region includes 11–154 amino acids. Copper is located at the active site of SOD1. The superoxide dismutase activity of erythrocortin (and the major copper-containing proteins in almost all tissues) inhibits cytochrome c reduction by exogenous addition of superoxide or in situ production of superoxide [20]. This reaction constitutes the basis for the determination of enzymes that are still commonly used today.

Similar to the SOD1 protein, the SOD2 protein also has two functional domains. In addition to the Mn/Fe catalytic domain [1~198 aa], there is a mitochondrial transport peptide region of up to 24 amino acids in the amino and Mn/Fe catalytic domains. SOD2 is synthesized in the form of a precursor with a mitochondrial targeting signal peptide in cells and enters the mitochondrial matrix through its 2 kDa leader sequence. The peptide is then cleaved to produce mature and enzymatically active proteins that play a key role in mitochondria [15,21].

Precocious SOD3 contains four functional domains, namely extracellular secretion of amino-terminal signal peptide, a glycosylation domain [1–95 aa], a catalytic domain with active and Cu^2+^/Zn^2+^ binding sites [96–194 aa], and a heparin-binding domain (HBD) [195~222 aa]. SOD3 is a secreted protein containing a putative 18 amino acid signal peptide at the N-terminus that directs the enzyme specifically to extracellular space. The SOD3 protein is also composed of three functional domains. The amino terminal residue [1–95 aa] contains an N-glycosylation site at Asn-89, which facilitates the separation of SOD3 from cytoplasmic SOD1 and greatly improves the solubility of the protein; the amino-terminal residue [96–194 aa] contains the active site and has about 50% homology with SOD1; the aminoterminal residue [195–222 aa] contains a C-terminal region corresponding to the heparin-binding domain (HBD), which has a positively charged residue cluster that is essential for binding to heparin sulfate proteoglycans.

Although the HBD of SOD3 does not participate in the enzymatic activity of SOD3, its HBD region is required for binding to type I collagen and LDL receptor-associated proteins, which are absorbed and internalized into cells via adhesion proteins and low-density protein receptor-associated proteins (LRP)-mediated endocytosis pathways.

Although the HBD of SOD3 does not participate in the enzyme activity of SOD3, its HBD region is required for binding to type I collagen and low-density lipoprotein receptor-associated protein, which is taken up and internalized into cells via adhesion proteins and low-density protein receptor-associated proteins (LRP)-mediated endocytosis pathways. An existing literature has confirmed that HBD is involved in nuclear translocation and endocytosis [22], but its molecular mechanism is still unclear. Therefore, it is necessary to further explore the molecular mechanism of SOD3 HBD regulating intracellular uptake.

### 2.3. SODs Catalyze the Reaction and Conversion of Superoxides

**Figure 2:**
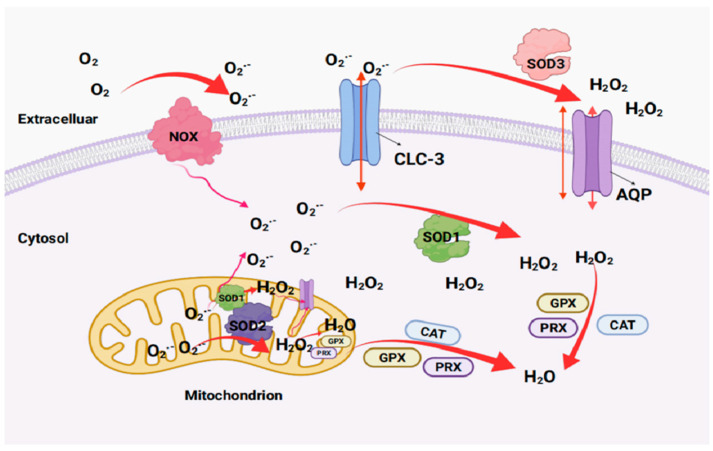
The reaction of superoxide catalyzed by SODs (created by Biorender). GPX: glutathione peroxidase; PRX: peroxiredoxin; CLC-3: Cl-channel-3.

Intracellular and extracellular O_2_^•−^ is produced by plasma membrane-bound NADPH oxidase (NOX). SOD1 and SOD2 convert intracellular superoxide to H_2_O_2_ and oxygen molecules, while SOD3 converts extracellular superoxide to H_2_O_2_ and oxygen molecules. Extracellular H_2_O_2_ is transported to cells through the aquaporin (AQP) channel and converted to water by the activity of catalase (CAT), peroxidase (PRX) and glutathione peroxidase (GPX) [23]. Extracellular O_2_^•−^ can enter cells through Cl-channel-3 (CLC-3) channels and be oxidized and disproportioned by SOD1 or SOD2 to produce H_2_O_2_ and water [24] (Figure 2).

## 3. SOD Gene Expression and Regulatory Mechanism

**Figure 3:**
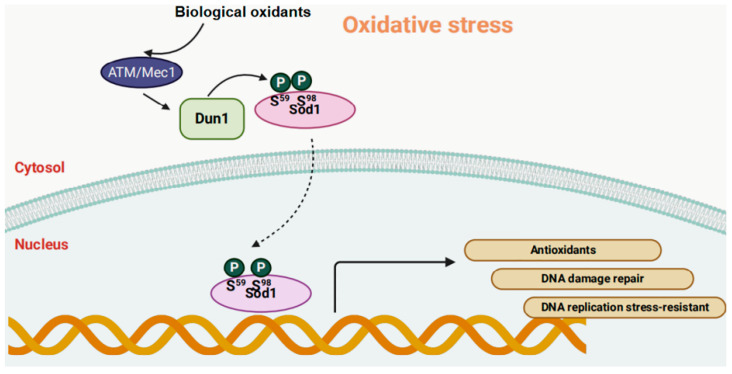
SOD1 acts as a nuclear transcription factor to regulate oxidative stress resistance (created by Biorender). ATM: ataxia-telangiectasia mutated; Mec1: mitosis entry checkpoint 1; Dun1: DNA damage checkpoint kinase.

Deletion of SOD1 in yeast and mice leads to widespread cell and genomic DNA oxidative damage [25,26]. It has been found that H_2_O_2_ is a key biological oxidant signal that controls the nuclear translocation of SOD1 to prevent oxidative damage to the genome. As the level of H_2_O_2_ increases, SOD1 enters the cell nucleus. Biological oxidants promote the binding of SOD1 with the effector kinases Mec1 (Mitosis entry checkpoint 1)/ataxia-telangiectasia mutated (ATM) (ATM kinase is known as an oxidation sensor directly activated by hydrogen peroxide) and Cds1/Dun1, as well as the phosphorylation of Dun1 at S^59^ and S^98^ sites on SOD1, promoting the nuclear localization of SOD1 [26,27]. Nuclear SOD1 regulates the expression of a large number of oxidative response genes, thereby promoting resistance to oxidative stress and DNA damage repair and replication stress relief (Figure 3). Numerous studies have found that impaired SOD1 activity in cataract lenses might be caused by genetic polymorphisms in the SOD1 gene, mutations in the coding and non-coding regions, and epigenetic regulation through promoter modification [28,29,30]. In age-related cataracts, histone 3 and histone 4 undergo deacetylation at −600 bp of the SOD1 promoter in the cataract lens, with low acetylation at −1500, −1200, and −900 bp [31]. Decreased acetylation of the SOD1 promoter histone leads to a decrease in SOD1 expression. This confirms that histone acetylation plays an important role in regulating SOD1 expression and the pathogenesis of age-related cataracts.

SOD2 is encoded by nuclear genes and is initially transcribed in the cytoplasm as a larger precursor. Subsequently, it binds to transport stimulators on the mitochondrial membrane by signal peptides, undergoes mitochondrial treatment to remove N-terminal lead peptides, and becomes a mature protein. Finally, it is localized as an active homotetramer in the mitochondrial matrix. The gene structure of SOD2 includes three important regulatory regions, namely the GC-rich promoter adjacent to the transcription start site, the enhancer in the second intron (~1900 bp), and highly conserved upstream regulatory elements (~800–1500 bp) in eukaryotes. These regions dynamically regulate gene expression in response to transcription factors, inflammatory factors, and drug treatments. Additionally, the expression of SOD2 genes is regulated by epigenetic factors, and the binding sequences of transcription factors such as transcription factor Sp1, activated protein-2 (AP-2), nuclear factor-κB (NF-κB), cyclic cAMP-reactive electron binding protein (CREB), and forkhead box class O proteins (FOXOs) are present in its promoter region. Studies have shown that inducible response elements like the tet response element (TRE) may play an important role in the regulation of Mn-SOD gene expression [32,33]. Borrello et al. found that manganese deficiency in mice negatively regulates Mn-SOD transcription levels, resulting in reduced liver manganese content, hepatic Mn-SOD enzyme activity, and mRNA levels [34]. Moreover, the SOD2 expression product requires selective modifications and sorting for proper functioning. For example, when the mitochondrial targeting sequence is lacking, the expressed polypeptide cannot enter the mitochondria and accumulates in the cytoplasm in an inactive state. Furthermore, the level of MnSOD is directly related to the acetylation state of histones in SOD2. It was found that the decrease of MnSOD expression in breast cancer cell lines was related to the deacetylation of H3K9 and hypomethylation of H3K4 on the seven transcriptional regulatory elements in SOD2 [35]. Similarly, in breast cancer, it was also confirmed that the expression of MnSOD can be inhibited by histone deacetylation and hypomethylation, thus forming a closed chromatin structure at SOD2, inhibiting the function of transcription factors [36].

**Figure 4:**
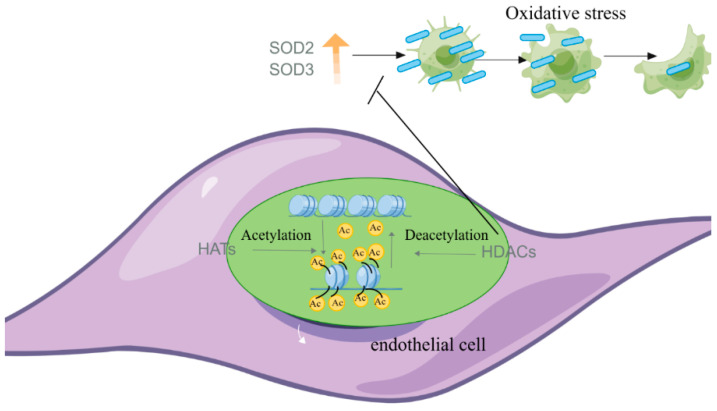
Effects of HAT and HDAC on endothelial cells (EC) related to vascular function (by Figdraw).

The transcription of the SOD3 gene is regulated by epigenetic modifications in the promoter region, including DNA methylation and histone acetylation/deacetylation. As a result, the protein could only be detected in a limited number of cell types, such as lung fibroblasts, epithelial cells, and vascular smooth muscle cells. Studies have shown that histone acetylation is associated with active transcription [37,38]. Mechanistically, the addition of negatively charged acetyl groups to specific lysine residues in histones could reduce the electrostatic affinity between histones and DNA, disrupting their interaction and leading to chromatin relaxation, thereby activating gene transcription. The intracellular levels of SOD3 are tightly regulated by histone acetyltransferases (HATs) and histone deacetylases (HDACs). Inhibition of HDAC3 significantly promoted SOD3 expression and benefited the alleviation of idiopathic pulmonary arterial hypertension (IPAH) [39]. Inhibitors of HDAC3 could also increase SOD levels by inhibiting the nuclear translocation of HDAC3 [39]. However, it is unclear whether HDAC3-induced SOD increases are a direct effect of HDAC3 activity or the result of inflammatory inhibition, which requires further research to investigate this (Figure 4).

## 4. The Application and Research Status of SODs in Medicine, Food, and Cosmetics

### 4.1. Application of SODs in Medical Treatment

**Figure 5:**
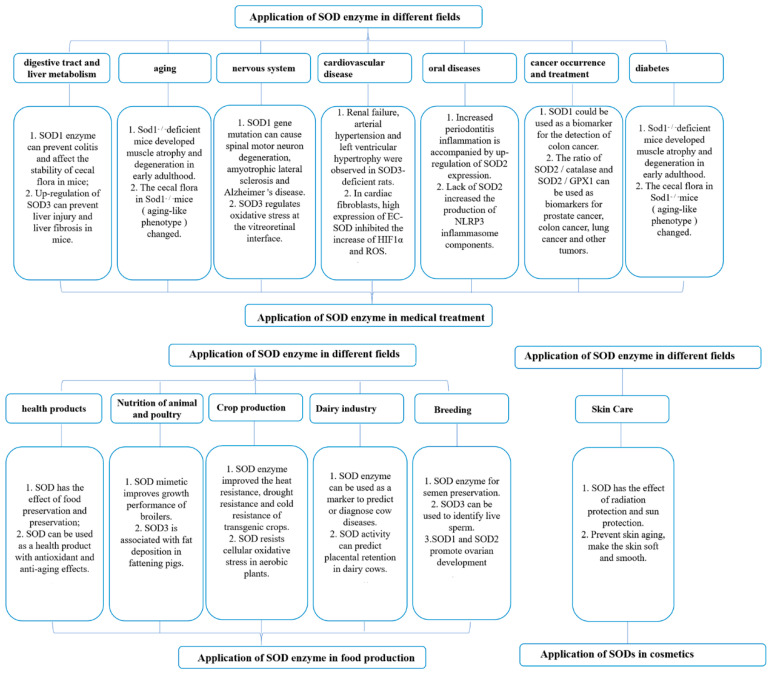
Application of SODs in medical, food, and skin care products.

The research of SODs in medicine has been widely reported, such as the treatment of diabetes, cancer, brain injury, and other diseases [40,41,42]. The incidence of diabetes is increasing exponentially worldwide, and one of the main mechanisms by which complications of diabetes develop is oxidative stress [40]. Oxidative stress is considered to be an important upstream event for the development of diabetic complications and insulin resistance, induces pathophysiological molecular mechanisms, and initiates a cascade of insulin resistance and diabetes [43]. In diagnosing or treating diabetes, a study found that increased levels of SOD1 protein helped reduce the apoptosis induced by maternal diabetes. In a separate study, it was confirmed that maternal diabetes inhibited SOD2 expression while exacerbating autism in offspring induced by oxidative stress in mice. These studies suggested that SOD1 or SOD2 might be used as a target for diagnosis or treatment in the development and treatment of diabetes. Cancer cells are highly metabolically active and hypoxic cells that tend to produce increased oxidative stress due to massive growth and inadequate vascular flushing. Oxidative stress disrupts DNA through mitochondrial membrane diffusion. The production of O_2_^•−^, H_2_O_2_, and HO• activates tumor signaling, enhances cell survival and proliferation, and drives DNA damage and genetic instability [43]. In the diagnosis or treatment of cancer, SOD1 gene mutations caused paralysis in mice, promoting tumor proliferation and invasion. Furthermore, SOD2 acetylation promoted cancer progression in mice. These findings revealed that SOD1 and SOD2 could be used as a target for diagnosis or treatment in the onset and treatment of cancer. After mechanical damage to the brain (secondary injury), the release of excitatory amino acids such as glutamate causes excessive stimulation of the N-methyl-D-aspartate (NMDA) receptor. The release of high levels of Ca^2+^ within cells activates proteases, lipases, and endonucleases, causing protein degradation, phosphorylation damage, and more. The increased Ca^2+^ in cell mitochondria inhibits the production of stimulated oxidants through pathways such as cytochrome c release and respiratory chain [44,45]. In the diagnosis or treatment of diseases such as brain injury, reducing the level of SOD2 protein in mouse hippocampal neurons can reduce brain damage caused by hypoxia/reoxygenation. Similarly, SOD3 reduces obesity and inflammation caused by high fat, as well as brain damage due to cerebral ischemia [46,47,48,49]. These suggested that SOD2 or SOD3 could serve as important reference indicators for brain injury. It could be seen that SODs play an important role in the diagnosis or treatment of diabetes, cancer, brain damage, and other diseases caused by oxidative stress. In the future development process, more and more SOD medical value will continue to be explored and discovered. The research of SODs in medical, food, and skin care products is shown in Figure 5.

#### 4.1.1. Management of SODs in Digestive Tract and Liver Metabolism

Gastrointestinal diseases have always been valued by researchers, doctors, and patients, and more researchers have focused on the regulatory role of SODs in the gastrointestinal tract. For example, knocking out the SOD1 gene in mice resulted in weight loss, disruption of the epithelial cell barrier, and decreased antioxidant enzyme activity [50]. In addition, the SOD1 enzyme prevented the occurrence of colitis and affected the stability of cecal flora in mice. Dextran sodium sulfate-induced SOD1 knockout colitis mice increased the levels of neutrophils, monocytes, pro-inflammatory CD11c^+^ macrophages, and CD11b^+^CD103^−^ dendritic cells and decreased the levels of anti-inflammatory CD206^+^ macrophages and CD11b^−^CD103^+^ dendritic cells [50]. One study found that the expression level of SOD3 was downregulated in the mouse liver fibrosis model induced by carbon tetrachloride (CCL). Downregulation of SOD3 led to spontaneous liver injury and fibrosis in mice, increased collagen deposition, and aggravated CCL-induced liver injury [51]. A published study suggested that injection of a SOD1 enzyme produced by Bacillus amyloliquefaciens into dextransulfatesodium (DSS)-treated SOD1 knockout mice could prevent colitis by inhibiting p38 mitogen-activated protein kinase (MAPK)/NF-κB signaling [50]. Based on the effect of SODs on the prevention of colitis, it is hypothesized that SODs can be used as a diagnostic marker in the prevention of colitis.

#### 4.1.2. SODs Management of Aging

Researchers are trying to find health products or drugs to meet most people’s dreams of pursuing health and youth, and the efficacy, mechanism of action, and promotion and application of SODs in anti-aging have been explored and developed. Mitochondrial reactive oxygen species production, oxidative stress, decreased function, and apoptosis are considered core components of the aging process, accompanied by muscle mitochondrial dysfunction and corresponding changes in the gastrointestinal flora.

A study found that the expression levels of SOD3 in the skin tissues of aging mice and humans were significantly lower than those in young skin tissues, while the expression levels of SOD1 and SOD2 remained unchanged during the aging process [52]. Furthermore, Su et al. showed evidence that Cu/Zn-superoxide dismutase (SOD, Sod1^−/−^)-deficient mice showed muscle atrophy, weakness, and neuromuscular junction (NMJ) degeneration in early adulthood, similar to normal muscle aging, and the transgenic expression of SOD1 specifically prevented muscle mitochondrial function and calcium treatment defects in neurons of SOD1-deficient mice [53]. Published literature revealed that mitochondria from heterozygous (SOD2^+/−^) mice were partially deficient in MnSOD, showing increased proton leakage, respiratory depression, and mitochondrial oxidative damage, while liver mitochondria from homozygous mutant mice were completely deficient in MnSOD, showing significant respiratory depression and significant sensitization of mitochondrial permeability transition pores [54]. The evidence demonstrated that the treatment of foreskin fibroblasts with recombinant SOD3 reduced intracellular biological oxidant levels and MMP-1 secretion while increasing type I collagen secretion. In addition, in vitro experiments confirmed that TNF-α-induced fibroblasts promoted the increase in SOD3 activity. On the one hand, it reduced the expression of MMP-1 mRNA by inhibiting the phosphorylation levels of NF-kB, p38 MAPK, extracellular regulated protein kinases (ERK), and c-Jun N-terminal kinase (JNK). On the other hand, high expression of SOD increased the expression of collagen type I alpha 1(COL1A1) and COL1A2 mRNA in collagen fibers [51]. Compared with SOD1^+/+^ mice (wild type), mice lacking SOD1 (SOD1^−/−^) (senescence-like phenotype) had a significant increase in *Paraprevotella*, *Prevotella*, *Ruminococcus,* and *Bacteroides* but a significant decrease in *Lactobacillus* [55]. According to the decrease of SOD3 expression in aging mice and human skin tissues, SOD1-deficient mice showing muscle aging in early adulthood, mice lacking SOD2 in mitochondria partially showing proton leakage, respiratory depression and increased mitochondrial oxidative damage, etc., all confirmed that SODs are indispensable in anti-aging.

#### 4.1.3. SODs Management of the Nervous System

Different types of SODs play an important role in improving sports injury and repairing diabetic vitreoretinopathy and other diseases. In terms of improving sports injury, one study suggested that a large number of mutations in the mouse SOD1 gene caused spinal motor neuron degeneration and progressive motor defects, leading to paralysis [55]. Human SOD1 gene mutation was closely related to the pathogenesis of neurodegenerative diseases such as amyotrophic lateral sclerosis and Alzheimer’s disease [56,57]. It was found that the adenovirus vector overexpressing SOD3 was directly injected into the paws of neutrophil cytosolic factor 1(Ncf1) (∗/∗) mice with collagen-induced arthritis [58]. The results demonstrated that SOD3 reduced the severity of arthritis in Ncf1 (∗/∗) mice and wild-type mice, emphasizing the important role of SOD3 in relieving arthritis in mice. The high expression of SOD3 significantly reduced the expression of the pro-apoptotic gene Bax and increased the expression of the anti-apoptotic gene Bcl-2, which alleviated ischemic stroke in rats by improving apoptosis [58]. A published article confirmed that no increase in SOD2 expression was detected in primary neurons or astrocytes, while the mRNA and protein levels of SOD2 were significantly increased in rat primary microglia treated with LPS, poly (I:C), peptidoglycan, or CpG oligodeoxynucleotides, suggesting that SOD2 was specifically induced in microglia under inflammatory conditions [59]. When the mRNA and protein levels of SOD2 increased, the activity of NF-κB was inhibited, thereby reducing the expression of pro-inflammatory cytokines [59]. In the repair of retinal diseases, evidence has confirmed that the increase in biological oxidants in the vitreous eye was related to the pathogenesis of diabetic vitreoretinopathy (DVR) [60]. A study revealed that SOD3 is differentially located in the vitreous sub-structure through specific proteoglycan interactions to prevent oxidative damage to the adjacent neuroretina, indicating that SOD3 could help regulate oxidative stress at the vitreoretinal interface [61]. These studies have confirmed that different types of SOD enzymes also play an indispensable role in neurological diseases.

#### 4.1.4. SODs Management of Cardiovascular Disease

The application of SODs in cardiovascular diseases is also very common, and its mechanism of action has been widely reported. Under the condition of hypoxia, free radicals increase greatly, which promotes the activation of cardiac fibrosis marker snail. Subsequently, the activity of methylase increased, the expression of tumor suppressor Ras association domain family 1A (RASSF1A) was downregulated, and the ERK1/2 signaling pathway was activated, which regulated the activation of cardiac fibroblast proliferation and the transformation of endothelial cells into myofibroblasts [22]. On the one hand, high expression of EC-SOD inhibited the increase in methylase activity and downregulated the expression of recombinant DNA methyltransferase 1(DNMT1) and DNMT-3b, thereby inhibiting the increase in HIF1α and biological oxidants. Another benefit of antioxidants was the chelation of hydrogen peroxide, which significantly reduced the activation of ERK1/2, both of which could counteract the oxidative stress associated with the pathogenesis of these cardiovascular diseases [62]. In addition, SOD3-deficient rats developed severe renal failure, arterial hypertension, and left ventricular hypertrophy at 21 weeks of age [62]. Therefore, it can be seen that SODs have the effect of regulating heart function, kidney function, and arterial hypertension in cardiovascular diseases.

#### 4.1.5. SODs Management of Oral Diseases

Periodontitis is a destructive disease that invades the alveolar bone, gingiva, periodontal ligament, and cementum [63]. When the inflammatory signal of periodontitis was enhanced or prolonged, SOD2 expression was upregulated by activating the NF-κB signaling pathway [64]. Gene manipulation of SOD2 by clustered regularly interspaced short palindromic repeats (CRISPR)/CRISPR associated 2 (Cas2) system showed that the lack of SOD2 increased the production of NOD-like receptor thermal protein domain associated protein 3 (NLRP3) inflammasome components [65], suggesting that intracellular SOD2 has a protective effect by inhibiting the NLRP inflammasome-cysteinyl aspartate-1-IL-1β axis under inflammatory conditions. This evidence verified that SOD also plays a vital role in oral inflammation.

#### 4.1.6. SODs Management of Cancer Occurrence and Treatment

Studies of SOD1, SOD2, or SOD3 in colorectal, lung, and other cancers have also been widely reported [66,67]. The upregulation of SOD1 in fibroblasts promoted the progression of breast cancer [67]. Inhibition of SOD1 selectively promoted cancer cell apoptosis by regulating biological oxidant signaling networks [68]. The function of SOD2 as a tumor suppressor or promoter was closely related to its function as a mitochondrial oxidant regulator, which played a cytoprotective role by scavenging harmful oxygen free radicals in the main cell metabolic centers. Reduction or loss of SOD2 expression is thought to affect cell cycle progression by increasing biological oxidant-mediated DNA damage [15]. Reduction or loss of SOD2 expression was thought to affect the progression of the cell cycle by increasing oxidant-mediated DNA damage.

Acetylated SOD2 promoted hypoxia signal transduction by increasing mitochondrial activity and promoting reprogramming of breast cancer stem cells [69]. SOD3, as an antioxidant immobilized in the extracellular space, was used to protect cells and biomolecules from superoxide-induced damage and to protect the biological activity of nitric oxide by inhibiting the diffusion restriction reaction between NO and superoxide. SOD3 was involved in the defense of oxidative stress and the prevention of estrogen-mediated breast cancer [46].

A study has shown that SOD2/catalase and SOD2/GPX1 ratio could be used as biomarkers for prostate cancer, colon cancer, lung cancer, and other tumors [70]. In terms of carcinogenesis, it was found that the increase in SOD2 expression in mouse lung adenocarcinoma induced by Aflatoxin G1 (AFG1) was related to the upregulation of Vimentin, α-SMA, Twist1, and MMP. In addition, in vitro experiments confirmed that AFG1 induced high expression of TNF-α and SOD-2 in human macrophage THP-1 (MΦ-THP-1 cells); that is, AFG1 could induce a tumor-associated inflammatory microenvironment [71].

In terms of cancer diagnosis, the SOD1 level might be used as a valuable biomarker for the detection of human colon cancer. For people aged 30 to 49 years in the second grade of colorectal cancer (CRC), the SOD1 content in serum reached 1240.7 U/mL. Additionally, in the third grade, the SOD content of the population reached 2428.0 U/mL, and the SOD content of the fourth grade of the population reached 3832.8 U/mL [72]. Therefore, it was believed that the increase in CRC grade was proportional to the increase in the production of SOD1 as a human anti-cancer antioxidant.

In cancer treatment, the poor drug delivery of chemotherapy to tumor cells was partly due to the high permeability of tumor blood vessels. It was found that the re-expression of specific SOD3 in tumor-associated endothelial cells (ECs) increased the delivery of doxorubicin (Doxo) and the effect of chemotherapy on tumors. On the one hand, the increase in SOD3 activity promoted the accumulation of nitric oxide around blood vessels and reduced vascular leakage by inducing the transcription of vascular endothelial cadherin (VEC). On the other hand, SOD3 reduced the activity of HIF prolyl hydroxylase domain protein, increased the stability of hypoxia-inducible factor-2α (HIF-2α), and enhanced its binding to specific VEC promoter regions [73]. Thus, it can be considered that SOD1, SOD2, or SOD3 play an important role in colorectal cancer, lung adenocarcinoma, and other cancers.

#### 4.1.7. SODs Management of Diabetes

Diabetes is a relatively common disease and is hereditary. More and more evidence revealed that SOD enzymes play an important role in the prevention and treatment of diabetes [74,75]. For example, SOD1 ameliorated maternal diabetes-induced apoptosis by restoring the Wnt signaling pathway [15]. Maternal diabetes induced immune dysfunction in autistic offspring through SOD2 inhibition and oxidative stress in hematopoietic stem cells [6]. Maternal diabetes induced autism-like behavior through hyperglycemia-mediated sustained oxidative stress and inhibition of SOD2 [20]. The SOD2 enzyme alleviated obesity, inflammation, and insulin resistance induced by high-fat diet [21].

SOD3 was upregulated in the serum and placenta of physically active pregnant women [76]. One study confirmed that maternal exercise improves the metabolic health of offspring by increasing the expression and secretion of placental SOD3 mediated by vitamin D receptors [76]. SOD3 activated the AMP-activated protein kinase (AMPK)/ten-eleven translocation (TET) signaling axis in the liver of fetal offspring, leading to DNA demethylation of glucose metabolism gene promoters, enhancing liver function, and improving glucose tolerance [76]. It was indicated that the discovery of maternal exercise-induced crosstalk between placental-derived SOD3 and offspring liver provided a central mechanism for improving the metabolic health of offspring. The study found that a high-fat diet (HFD) induced oxidative stress in rats, after administering purple corn anthocyanin extract (PCAE) treatment by reducing plasma and liver malondialdehyde (MDA), plasma and liver lipid peroxidation index, liver weight, liver index and liver mitochondria SOD, improving plasma and liver total antioxidant capacity and liver SOD activity to improve oxidative stress [77]. This indicated that purple corn anthocyanin extract improves oxidative stress in high-fat diet rats through the superoxide dismutase mechanism. Indole-3-acetic acid (IAA) was an intestinal microbiome derived from the metabolites of dietary tryptophan. It reduced HFD-induced hepatotoxicity in mice by improving insulin resistance, lipid metabolism, oxidation (biological oxidants and MDA levels and SOD activity in liver tissue), and inflammatory stress [78]. Indeed, SODs play an indispensable role in improving diabetes by lipid metabolism.

### 4.2. Application of SODs in Food Production

#### 4.2.1. Application in Health Products

SOD is widely found in animals, plants, and microorganisms. As a specific scavenger of superoxide anion free radicals and a high-quality regulator of various functions of the human body, it has been widely used in food production applications [79]. The application of SOD in food mainly has the following two aspects. On the one hand, researchers confirmed the effectiveness of oral SODs, so SODs could be directly used as a food additive, and have developed many functional foods rich in superoxide dismutase, such as SOD longevity wine, SOD mayonnaise, SOD milk candy, etc. [80]. On the other hand, the fresh-keeping effect of SOD might produce a large amount of O_2_^•−^ in the process of food production and transportation. These O_2_^•−^ easily react with nutrients in food such as ascorbic acid and tocopherol. As an antioxidant, superoxide dismutase could prevent the degradation of food quality due to peroxidation and has a good antioxidant effect. Therefore, SOD is called a food preservative [79]. China had implemented the standard requirements of SOD health products (functional foods), established the SOD food market mechanism, and encouraged the development of reasonable SOD applications, which had great room for development in the food industry.

#### 4.2.2. Nutrition of Animal and Poultry

As a feed additive, the SOD enzyme is used in livestock and poultry feed to improve the utilization rate of animal nutrition and enhance immunity. The addition of different levels of SOD mimetics in the diet had no significant effect on the daily feed intake, average daily gain, feed-to-gain ratio, PH, redness, and yellowness of breast muscle of broilers, but the activity of SOD in serum increased, and the content of T-AOC and MDA decreased [81]. Adding 300 mg/kg resveratrol to the diet of finishing pigs could reduce the level of malondialdehyde in the longissimus muscle and increase the activity of SOD and SOD2 mRNA levels [82]. The results revealed that the lipid peroxidation level of delayed-feeding chicks was higher than that of free-feeding chicks (free-feeding after hatching), and the mRNA expression of Cu/Zn-SOD, Mn-SOD and GPX7 in delayed-feeding chicks (delayed-feeding chicks from 0 to 2 days of age) was lower than that of free-feeding chicks, but there was no difference in catalase mRNA level [83]. This indicated that delayed post-incubation-feeding affected the lipid peroxidation of muscle during the growth of chicks. The results showed that the supplementation of 40 mg/kg genistein in the diet of laying hens could upregulate the expression level of the SOD3 gene in the mother and increase the percentage of chest muscle and liver index of chicks [84]. More and more studies have confirmed that the antioxidant effect of SODs is mediated by biological oxidants [85,86]. As a research hotspot in livestock and poultry production, oxidative stress could provide a theoretical basis for the solution of nutritional metabolic diseases in livestock and poultry production. The SOD3 gene is located on pig chromosome 8 and is associated with fat deposition in pigs [87]. Similarly, the result in humans showed that the degree of methylation of SOD3 was associated with fat phenotypic traits [88].

#### 4.2.3. Dairy Industry

SOD is an important antioxidant enzyme which has different physiological functions and can be used as a nutritional enhancer in food. The yield of SOD has become a high-quality evaluation index for yogurt fermentation. It was found that the yield of SOD of *Lactobacillus plantarum subsp. plantarum* F8 was 2476.21 ± 1.52 U g^−1^, which was used as a probiotic for yogurt fermentation. The addition of this strain made the SOD concentration of fermented yogurt 19.827 ± 0.323 U mL^−1^, which was 63.01, 50.11, and 146.79% higher than that of Guangming Dairy Yogurt 1911, Junlebao, and Nanjing Weigang, respectively [41]. In addition, the addition of this strain upregulated 12 non-volatile metabolites in yogurt, and the addition of millet enzymatic fermentation increased the content of non-volatile metabolites in yogurt.

In fact, the SOD enzyme can be used as a marker for prediction or diagnosis in the prevention or diagnosis of cow diseases. The most important factors of metabolic and reproductive diseases in dairy cows occur after the increase in oxidants and the formation of oxidative stress. A published study showed that the concentrations of superoxide dismutase (*p* = 0.04) and catalase (*p* = 0.08) in subclinical ketosis cows were lower than those in healthy cows, and the concentration of monohydroxybutyric acid (BHBA) in pregnant cows was significantly lower than that in non-pregnant cows (*p* = 0.008) [89]. It was suggested that oxidative stress biomarkers are promising biomarkers for predicting subclinical ketosis in postpartum dairy cows. Furthermore, flow cytometry was used to evaluate neutrophil phagocytosis and oxidative burst activity in the peripheral blood of dairy cows with or without placental retention at 10 ± 4 days before or 10 ± 4 days after delivery, but there was no significant difference in neutrophil phagocytosis and oxidative burst activity, phagocytosis and oxidative fluorescence intensity, and phagocytosis and oxidation index. However, the study found that the serum SOD activity of cows with placental retention and healthy cows 10 ± 4 days before delivery was 30 ± 4 IU/mL and 17 ± 2 IU/mL, respectively (*p* ≤ 0.05) [90]. It was suggested that the increase in prenatal SOD activity might be an inducing factor and can be used as a predictive biomarker for placental retention.

#### 4.2.4. Breeding

Antioxidants are often used in the process of animal semen preservation because they can prevent the decrease of sperm motility and mitochondrial membrane potential and protect sperm from apoptosis so as to ensure the quality of semen. A study confirmed that the addition of 200 U/mL SOD diluent to the semen of Qingjiao Ma chicken cocks caused an increase in sperm motility, and the SOD group had higher plasma membrane integrity after thawing, and less sperm showed apoptosis [91]. Therefore, SOD as an antioxidant can be recommended as an additive component of chicken semen freezing diluent. In addition, SOD3 also plays an important role in the identification of live sperm. For example, lactoferrin in horse semen was closely bound to SOD3, and the protein complex was selectively bound to non-viable sperm, which might be labeled by phagocytosis of neutrophils to identify live sperm and dead sperm [92]. In addition, SOD1 and SOD2 also play an important role in ovarian development. One study revealed that histological analysis of the ovaries of SOD1-deficient mice showed many primary and small antral follicles but rarely the corpus luteum. In addition, ovaries from postnatal SOD2-deficient mice transplanted into the wild-type host’s bursa showed all stages of folliculogenesis, including the corpus luteum, and could produce viable offspring [93]. It was explained that SOD1 plays an important role in female reproductive function, while SOD2 is not necessary for ovarian function.

#### 4.2.5. Crop Production

Superoxide dismutase, as a general gene of agricultural biotechnology, plays an irreplaceable role in the process of crop production. It was found that overexpression of wheat mitochondrial Mn-SOD in transgenic oilseed rape improved the heat tolerance, drought resistance, and cold resistance of plants under field and artificial drought stress [94]. It was also confirmed in potato breeding that the lines transformed with SOD3.1 had greater freezing tolerance than the parental lines, which was helpful in producing significant economic benefits in high-value/stress-sensitive crops such as potatoes [95].

In aerobic organisms, SOD plays an important role in cellular defense against oxidative stress. In the production of cucumber (*Cucumis sativus* L.) fruit, the CuZnSOD cDNA (mSOD1) of cassava was introduced into cucumber fruit by Agrobacterium-mediated transformation using the ascorbic acid oxidase promoter with high expression in fruit [96]. Southern blot analysis confirmed that the mSOD1 gene had been correctly integrated into the nuclear genomes of the three cucumber plants, and the SOD-specific activity (unit/mg protein) in the transgenic fruits was about three times higher than that in the non-transgenic plants [95].

### 4.3. Application of SODs in Cosmetics

When human skin is in direct contact with oxygen, it will cause skin aging and damage [96]. SOD is a powerful oxygen-free radical scavenger, which has obvious sunscreen and radiation protection effects. At the same time, SOD is an anti-inflammatory enzyme with an obvious anti-inflammatory effect. Domestic and foreign scholars have conducted a lot of research on the toxicity of superoxide dismutase and proved that superoxide dismutase has no toxic side effects and is a very safe cosmetic. A study showed that the migration of Langerhans cells to lymph nodes was impaired in EC-SOD transgenic mice [97]. EC-SOD downregulated contact hypersensitivity by inhibiting inflammatory response, suggesting that EC-SOD may become a treatment for inflammatory skin diseases. At the same time, one researcher labeled SOD with iodine and applied it to the skin of mice. The results showed that it could penetrate into the skin and be absorbed well [98]. Moreover, a previous study revealed that pretreatment with superoxide dismutase and cell-penetrating peptide (TAT-SOD) in male subjects one hour before ultraviolet radiation b (UVB) radiation promoted an average minimum erythema dose increase of 36.6 ± 18.4% compared to carrier control, and TAT-SOD pretreatment reduced apoptotic sunburn cell formation by 47.6 ± 8.6% in all subjects. This confirms that local application of superoxide dismutase and cell-penetrating peptide (HIV-TAT) could significantly reduce male skin damage induced by ultraviolet radiation b (UVB) [99]. In addition, there was literature confirming that superoxide dismutase-loaded niosomes were prepared by the thin layer hydration method and delivered to the hair follicles of guinea pig skin, which were then infiltrated through synthetic membranes and guinea pig skin [100]. Therefore, the application of SOD as an additive added to daily chemical products is gradually widespread. In the field of industrial daily chemical application in China, SOD as an effective component has been successfully added to the production process of Dabao SOD honey, Meijiajing SOD toothpaste, and other commodities. It is a pioneer in the field of SOD application in China. It was also widely used in other countries as a result of these aspects. On the one hand, the application of SOD in skin care products is a cosmetic additive. SOD can effectively scavenge oxygen free radicals, prevent skin aging, make the skin soft and smooth, and promote the moderate crosslinking of collagen, stabilize collagen and elastin, and make the skin full of elasticity. Another aspect is to prevent and treat some skin diseases as an external coating agent, which is used to eliminate skin redness and swelling caused by verification, assist in sun protection, etc.

## 5. Problems and Research Progress of SODs Application

### 5.1. Stability of SOD Enzyme

Poor stability, short half-life in vivo, and easy hydrolysis by proteases are the shortcomings of superoxide dismutase. Therefore, it cannot be used for large-scale production in the food industry. In order to convert various sources of SOD into food, SOD itself and its carrier must be modified. On the one hand, many scholars have proposed to change the composition and molecular structure of SOD by chemical modification, point mutation, liposome modification, and microencapsulation [101,102,103,104] without changing its biological activity so as to improve its stability [105]. Various researchers have increased the persistence of SOD by cross-linking SOD or attaching polymer substances (including dextran, albumin, Ficoll, polyvinyl alcohol, or polyethylene glycol (PEG)). Somack et al. modified purified SOD with MW PEG (41,000–72,000 daltons) and retained 90–100% of the native SOD activity [106]; Chang et al. modified SOD with N, O-carboxymethyl chitosan heparin self-healing hydrogel, found that the hydrogel modified SOD effectively reduced the loss of enzyme activity during the modification process, and significantly accelerated the progress of wound healing in the early stage of trauma [107]; Kumar et al. obtained SOD with improved thermal stability by point mutation [108]; Marin et al. encapsulated manganese dioxide nanoparticles into LbL polymer microcapsules to produce a synthetic antioxidant microreactor, which effectively scavenged hydrogen peroxide (H_2_O_2_) from solutions in an in vitro model and protected cells from oxidative stress [109].

On the other hand, some scholars believe that you can also start from the source of SOD. At this stage, some thermally stable SOD can be obtained from thermophilic bacteria such as *Thermococcum* sp., *Aeromonas* sp., and *Thermomyces* sp. The thermostable Fe/Mn-SOD of Geobacillus strain PCH100 (GsSOD) isolated from glacial soil by Guleria et al. and GsSOD can tolerate 100 °C and 130 °C for 15 min and 5 min, respectively [110]; Shahi et al. cloned the novel thermophilic bacteria Cohnella sp. A01 (CaSOD) gene into pET-26b (+) expression vector, and optimized the expression of recombinant protein (rCaSOD) in E.coli BL21 (E3). rCaSOD showed catalytic activity in a very wide range of pH (6.0–10.0) and temperature (35–75 °C) and was stable in a wide pH range of 3.0 to 11.0 [111]; Dong et al. isolated a thermoacidophilic Alicyclobacillus HJ strain from a hot spring in Tengchong, Yunnan, China, identified a hypothetical gene encoding superoxide dismutase (AaSOD) in this strain, and found that the enzyme was highly stable at any specific temperature up to 80 °C. Furthermore, the enzyme was stable in a wide pH range (2.0–10.0) [112]. The modified SOD has a wide pH range and high-temperature resistance, which provides it with strong stability in an acidic gastric environment and hints at its incredible potential as a food additive and for medical use.

### 5.2. Permeability of SOD Enzyme

The application of SOD in the food industry and healthcare products has been controversial. As a biological macromolecule, how SOD enzyme enters the human body through the cell barrier, but also whether it can be absorbed and utilized by human gastric juice and epidermis, is still inconclusive. There is a lot of evidence confirming that in order to improve the value of SOD utilization, researchers have developed SOD mimics such as 2,2,6,6-Tetramethyl-4-[[5-(triphenylphosphonio)pentyl]oxy]-1-piperidinyloxy bromide (Mito-Tempol), manganese (III) meso-tetrakis(N-(2′-n-butoxyethyl) pyridinium-2-yl) porphyrin (MnTnBuOE-2-PyP5+), and MitoQ (mitoquinol mesylate) to improve the membrane permeability of SOD enzyme [113,114,115,116], liposome-encapsulated active SOD protein [113], and improve its own cell transmission ability. However, these two methods have limitations in clinical application. Patients are prone to nausea, vomiting, temporary hair loss, and other symptoms [117]. Furthermore, the protein transduction domain (PTD) transduction domain can also mediate SOD transduction [118]. Since the discovery of the PTD transduction domain, it has been widely used in the transmembrane transport of macromolecular substances. PTD can maintain the biological activity of macromolecular substances and has the characteristics of fast transduction speed and high efficiency. However, the PTD transduction domain may have the disadvantages of lack of cell specificity and short action time. Therefore, the membrane permeability of SOD and the persistence of enzyme action are still the main contents of future research, which is also a key in food and medicine.

## 6. Conclusions and Prospect

This review discusses the wide range of medicinal and industrial values of SODs. They reduce oxidative damage by scavenging free radicals, fight against aging, inhibit diseases such as cancer, strongly support the treatment of oxidative stress-related diseases, and become a promising alternative to prevent and treat diseases. In addition, this paper highlights the health benefits of SODs, which can be used to develop new drugs, new foods, and new skincare products to improve the recovery and stability of production.

## Figures and Tables

**Table 1:** Types and characteristics of SOD.

Types	Location	Metal Cofactors	Combination
SOD1 (Cu/ZnSOD)	Cytoplasm, mitochondrial membrane space, and others (nucleus, lysosomes, and peroxisomes)	Cu^2+^ (catalyzing type) Zn^2+^ (stable form)	The two subunits are mainly combined by hydrophobic and electrostatic interactions; copper and zinc form coordination bonds with the histidine side chain on the active site.
SOD2 (MnSOD)	Mitochondrial matrix	Mn^3+^ (catalyzing type)	The manganese ion is coordinated with three histidine side chains, an aspartic acid side chain, and a water molecule or hydroxyl group (depending on the oxidation state of manganese).
SOD3 (EC-SOD)	Extracellular matrix, cell surface, and extracellular fluid	Cu^2+^ (catalyzing type) Zn^2 +^ (stable form)	The active sites of Mn-SOD and Fe-SOD have the same type of amino acids coordinated with metal ions.

## Data Availability

Not applicable.
